# Identifying Factors Associated with Prolonged Postoperative Mechanical Ventilation in Preterm Infants Undergoing Patent Ductus Arteriosus Ligation Using Machine Learning and SHAP Analysis: A Large-Sample Single-Center Retrospective Analysis

**DOI:** 10.3390/jcm15134984

**Published:** 2026-06-26

**Authors:** Qiang Gao, Yu Cao, Xiwang Liu, Xicheng Zhang, Xucong Shi, Liyang Ying, Liping Shi, Taixiang Liu, Xiangming Fan

**Affiliations:** 1 Department of Cardiac Surgery & Heart Center, Children’s Hospital, Zhejiang University School of Medicine, National Clinical Research Center for Child and Adolescents’ Health and Diseases, Hangzhou 310052, China; 2 Clinical Data Center, Children’s Hospital, Zhejiang University School of Medicine, National Clinical Research Center for Child and Adolescents’ Health and Diseases, Hangzhou 310052, China; 3 Neonatology Intensive Care Unit, Children’s Hospital, Zhejiang University School of Medicine, National Clinical Research Center for Child and Adolescents’ Health and Diseases, Hangzhou 310052, China

**Keywords:** machine learning, mechanical ventilation, patent ductus arteriosus, risk factors, preterm infants

## Abstract

**Objectives:** To analyze factors associated with prolonged mechanical ventilation (MV) after patent ductus arteriosus (PDA) ligation in preterm infants and identify high-risk patients. **Methods:** A retrospective analysis (2021–2025) was conducted on preterm infants (≤32 weeks) who underwent PDA ligation. Demographic, preoperative and postoperative data were analyzed; machine learning and SHAP analysis identified associated factors. **Results:** A total of 271 infants (152 males, 119 females; median gestational age 27 weeks [23–32 weeks], median birth weight 920 g [470–2220 g]) were included. Based on postoperative MV duration ≤6 days or >6 days, 150 cases were assigned to the short MV group and 121 to the prolonged MV group. Significant differences were found between groups in terms of gestational age, birth weight, weight at surgery, PDA diameter, postoperative slowest heart rate, Respiratory Severity Score (RSS), rate of bidirectional PDA shunting, preoperative high-frequency ventilation use, post-ligation cardiac syndrome incidence, and rate of preoperative pulmonary hemorrhage/atelectasis (*p* < 0.05). RSS was identified as the most central predictor of prolonged postoperative MV duration. RSS > 4.5 was associated with a markedly elevated risk of MV >6 days (AUC = 0.865, sensitivity = 80.2%, specificity = 82.5%). In contrast, birth weight > 1500 g or post-ligation heart rate >135 bpm was correlated with shorter MV duration. **Conclusions:** High RSS, low birth weight, and slow postoperative heart rate are associated with prolonged postoperative MV. This may aid in identifying infants requiring closer perioperative monitoring.

## 1. Introduction

Patent ductus arteriosus (PDA) is common in preterm infants. The chronic left-to-right shunting associated with a hemodynamically significant PDA can lead to cardiac dysfunction and pulmonary edema, resulting in decreased lung compliance and necessitating mechanical ventilation (MV) to maintain adequate gas exchange. However, prolonged MV is associated with a range of complications [1]. Cyclooxygenase (COX) inhibitors, such as indomethacin or ibuprofen, are often the first-line treatment to promote PDA closure. For infants with contraindications to or failure of COX inhibitor therapy, surgical ligation is performed to close the ductus arteriosus, with the goal of facilitating earlier weaning from MV. Nevertheless, in clinical practice, considerable variability was found in the duration of postoperative MV among infants following PDA ligation. This study aims to investigate the potential factors associated with prolonged MV after PDA ligation in preterm infants, thereby providing a reference for clinical management.

## 2. Materials and Methods

### 2.1. Inclusion and Exclusion Criteria

We conducted a retrospective study of preterm infants who underwent PDA ligation at our hospital between 1 January 2021 and 8 May 2025. Adequate communication about conditions and treatment was conducted with all guardians upon admission. Surgical plans and risks were explained preoperatively, and informed consent was obtained. The inclusion criterion was preterm infants with a gestational age ≤ 32 weeks who underwent PDA ligation. The exclusion criteria were as follows: (1) presence of other cardiovascular malformations, except for a small atrial septal defect or ventricular septal defect; (2) congenital airway anomalies; (3) congenital anomalies significantly affecting pulmonary artery pressure (e.g., omphalocele); (4) genetic disorders; (5) cases not receiving MV preoperatively; (6) cases of death before weaning from MV postoperatively; (7) severe intraventricular hemorrhage (Grade III or higher).

### 2.2. PDA Treatment Strategy

At our institution, preterm infants diagnosed with PDA initially receive two standard courses of COX inhibitor therapy [2]. For infants with contraindications to COX inhibitor therapy, or those in whom the ductus arteriosus fails to close after treatment and exhibit significant clinical symptoms or require MV, if they have hemodynamically significant PDA (as defined by Adil Umut Zübarioğlu [3]), the decision on whether to perform surgical treatment is made after multidisciplinary consultation involving cardiac surgery, cardiology, neonatology intensive care unit (NICU), and other departments. The ligations were performed at the bedside in the NICU, and the preoperative ventilation mode remains unchanged during the surgery. The procedure is carried out via a left posterolateral thoracotomy. The PDA was closed either by double silk ligation or with a titanium clip, depending on the surgeon’s preference.

### 2.3. Data Collection and Definitions

Data including sex, gestational age, birth weight, age at surgery, weight at surgery, PDA diameter, PDA-to-aorta (PDA/AO) ratio, presence of preoperative pulmonary hemorrhage or atelectasis (PHA), and occurrence of necrotizing enterocolitis (NEC) were collected. Preoperative and postoperative ventilator settings and vasoactive medication use were recorded. Intraoperative changes in systolic and diastolic blood pressure immediately following ligation were also collected. The duration of postoperative MV was defined as the time from the end of surgery until extubation; if reintubation and MV were required, the total duration of MV episodes was summed. The slowest heart rate within the first 24 h post-ligation was recorded (for cases with sustained arrhythmias, data after treatment were documented).

The PDA diameter, ascending aorta diameter, and direction of ductal shunting were determined by a dedicated echocardiographer through a retrospective review of the last echocardiogram performed prior to ligation.

Preoperative high-frequency oscillatory ventilation (HFV) use was defined as the receipt of HFV within five days prior to surgery. Preoperative PHA positivity was defined as the occurrence of pulmonary hemorrhage or atelectasis within five days prior to surgery [4].

The Respiratory Severity Score (RSS), calculated as mean airway pressure (MAP) multiplied by fraction of inspired oxygen (FiO_2_), was determined using the highest ventilator settings recorded within the 24 h preceding surgery.

Post-ligation cardiac syndrome (PLCS) was defined according to the criteria established by Jain et al. [5]: the occurrence of hemodynamic instability accompanied by respiratory failure within 24 h after PDA ligation, in the absence of septic shock. PLCS was characterized by either: (1) the need for vasoactive support for hemodynamic stabilization within 24 h, or an increase in vasoactive medication dosage by more than 20% sustained for at least one hour; or (2) the need for HFV, or an increase in FiO_2_ or MAP by more than 20% compared to preoperative baseline, sustained for at least one hour within the first 24 h post-ligation.

### 2.4. Group Allocation

In a nationwide UK cohort, A Warnock et al. [6] reported a median postoperative MV duration of 5 days after PDA ligation in preterm infants overall, and 7 days among preoperatively intubated patients. Building on this finding, we dichotomized patients into a short MV group (≤6 days) and a prolonged MV group (>6 days) by postoperative MV duration. (As shown in Supplementary File S1, the reintubation rate differed significantly between the two groups.)

### 2.5. Statistical Analysis

Statistical analysis was performed using SPSS Statistics version 24 (IBM, Armonk, NY, USA). In the first phase, descriptive statistics were analyzed for all enrolled infants, including sex distribution, gestational age, birth weight, age at surgery, weight at surgery, PDA diameter, bidirectional PDA shunting, PDA/AO ratio, increase in systolic blood pressure and diastolic blood pressure, preoperative NEC incidence, HFV rate, PHA rate, RSS, postoperative PLCS rate, slowest heart rate, and duration of postoperative MV. In the second phase, the differences in each variable between the two groups were compared separately. Continuous variables were tested for normality using the Shapiro–Wilk test. Variables following a normal distribution were compared using the *t*-test, while those not following a normal distribution were compared using the Mann–Whitney U test. Categorical variables were analyzed using the chi-square test. A *p*-value < 0.05 was considered statistically significant. In the third phase, linear regression analysis was performed on all variables to identify non-collinear variables, followed by logistic regression analysis to determine the factors associated with the duration of postoperative MV. To identify non-collinear predictors, candidate variables from univariate analyses and clinically relevant variables were assessed for multicollinearity using Spearman correlation and variance inflation factors (VIFs). Variables with VIF > 5 were sequentially excluded based on clinical relevance. A receiver operating characteristic (ROC) curve was plotted to calculate the area under the curve (AUC), sensitivity, and specificity. In the fourth phase, baseline machine learning algorithms including Logistic Regression (LR), Random Forest (RF), Support Vector Machine (SVM), and Extreme Gradient Boosting (XGBoost) were adopted to construct the perioperative and early postoperative risk-stratification mode. A total of 20 candidate predictors were considered for modeling the following: sex, gestational age, birth weight, age at surgery, weight at surgery, PDA diameter, bidirectional shunting, PDA/AO ratio, pre- and post-ligation systolic and diastolic blood pressure, systolic and diastolic blood pressure increase, slowest heart rate, NEC, HFV, PLCS, RSS, and PHA. All machine learning analyses were evaluated using stratified 5-fold cross-validation. Hyperparameters were tuned using clinically constrained grids, and model calibration and decision-curve analyses were performed to assess potential clinical utility. The mean absolute value of the Shapley Additive Explanations (SHAP) values for each variable was calculated, and, combined with logistic regression analysis, the most valuable factors associated with prolonged postoperative MV after PDA ligation in preterm infants were finally identified.

## 3. Results

A total of 318 preterm infants underwent PDA ligation during the 5-year study period. After excluding three infants with concomitant ventricular septal defect requiring subsequent surgery, one infant with a subglottic polyp, four infants with omphalocele, three infants with chromosomal abnormalities, 24 infants who were not intubated preoperatively, eight infants who died or were discharged against medical advice before weaning from MV, and four infants with severe intraventricular hemorrhage, a total of 271 infants were included in the final analysis. The cohort comprised 152 males and 119 females. The remaining variables are presented in Table 1.

Compared to the short MV group (*n* = 150), infants in the prolonged MV group (*n* = 121) had significantly lower gestational age, birth weight, and weight at surgery; larger PDA diameter; slower slowest heart rate; and higher RSS, rates of bidirectional PDA shunting, HFV positivity, PLCS positivity, and PHA positivity. These differences were statistically significant (*p* < 0.05) (Table 2).

We performed collinearity analysis on all variables and ultimately identified six non-collinear variables: slowest heart rate, PHA, RSS, PLCS, PDA/AO ratio, and birth weight. Logistic regression analysis of these variables revealed that each one-point increase in RSS was associated with a 289% increase in the possibility of postoperative MV > 6 days. The presence of preoperative PHA or postoperative PLCS increased the possibility by 90% and 47%, respectively. Each 0.1 increase in the PDA/AO ratio was associated with an approximately 4.6% increase in possibility. After multiplying the protective factors by −1 for analysis, we found that each 100 g decrease in birth weight was associated with a 42.6% increase in possibility, and each one beat-per-minute decrease in the slowest heart rate was associated with an approximately 5.9% increase in possibility. The results of the logistic regression analysis are presented in Table 3.

**Figure 1:**
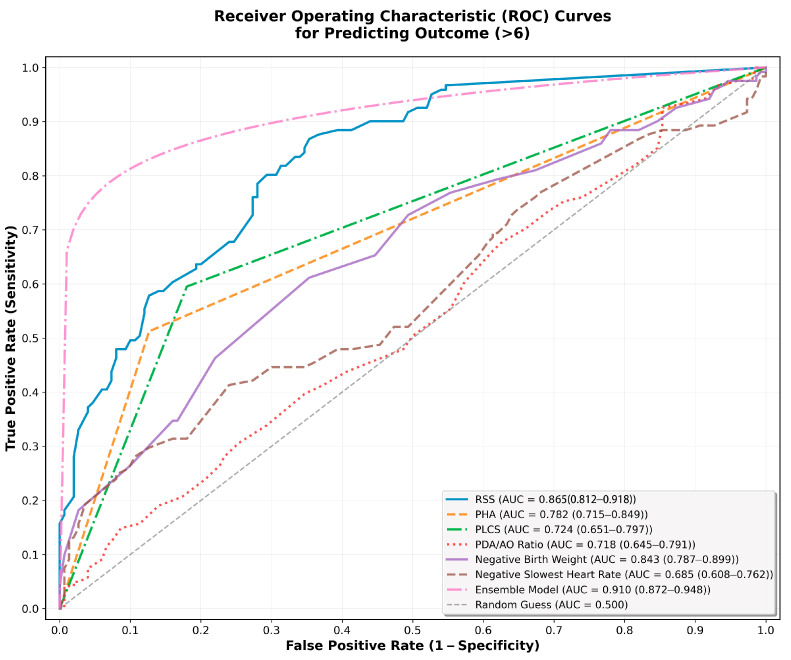
ROC curves for individual factors and the integrated model.

Univariate ROC analysis revealed that RSS and birth weight were the two strongest individual predictors, with AUCs (95% CI) of 0.865 (0.812–0.918) and 0.843 (0.787–0.899), respectively. An RSS value greater than 4.5 was associated with a significantly increased risk of postoperative MV duration >6 days, with a sensitivity of 80.2% and specificity of 82.5%. Similarly, a birth weight ≤1500 g was associated with a significantly increased risk, yielding a sensitivity of 81.5% and specificity of 79.8%. When six factors were combined into an integrated model, the AUC was higher than that of any other single factor (0.910 vs. 0.865), indicating improved predictive power (Table 4, Figure 1).

The ROC analysis identified 4.5 as the optimal cut-off value for RSS. RSS > 4.5 was defined as high RSS. After propensity score weighting, high RSS remained significantly associated with postoperative mechanical ventilation duration > 6 days (Table 5).

We evaluated the performance of four algorithms using stratified 5-fold cross-validation. XGBoost showed the highest mean AUC (0.901 ± 0.033), followed by RF (0.890 ± 0.029), LR (0.880 ± 0.032), and SVM (0.864 ± 0.023). The out-of-fold AUC and Brier score were also reported to avoid relying solely on discrimination (Table 6).

**Figure 2:**
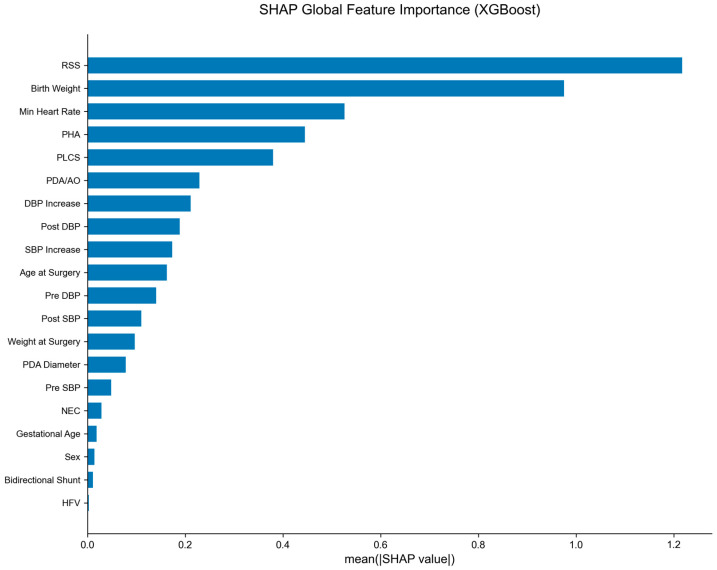
SHAP global importance analysis. The higher the SHAP value is, the greater its effect on the prolongation of MV is.

**Figure 3:**
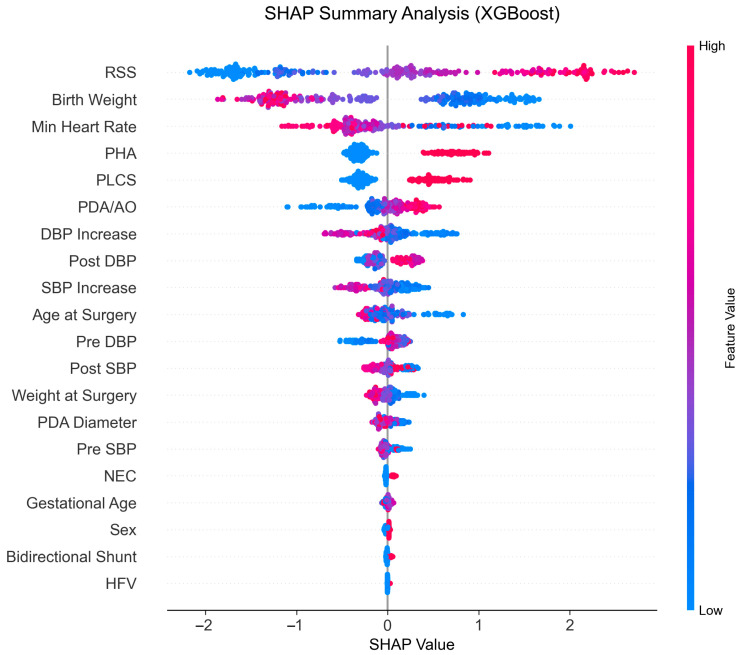
SHAP summary analysis. The larger the SHAP value is, the greater the impact on the prolongation of MV is. Feature values of influencing factors are marked in red when positively correlated with SHAP values and in blue when negatively correlated.

Analysis of the XGBoost model by calculating the mean absolute SHAP values for each feature revealed that the RSS was the most central predictor for prolonged MV, followed by birth weight and the slowest postoperative heart rate (Figure 2). RSS exerted a significant positive driving effect: high RSS (red data points) corresponded to positive SHAP values, markedly increasing the risk. Birth weight showed a negative driving effect, with low birth weight (blue data points), indicating a higher risk of difficult weaning in very low birth weight infants after surgery. Postoperative heart rate also exhibited a negative driving effect, with lower heart rates (blue points) tending to cluster in regions with SHAP values > 0, confirming that slow postoperative heart rate is associated with prolonged MV (Figure 3).

**Figure 4:**
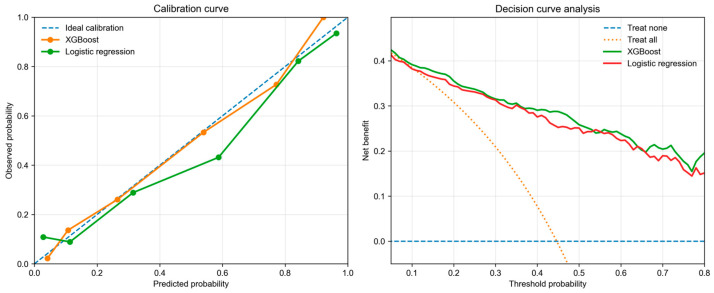
Calibration and decision curve analyses for XGBoost.

Calibration analysis and decision curve analysis for the XGBoost model revealed that it exhibited good discriminative ability, with predicted probabilities well consistent with actual risks. As a linear baseline model, logistic regression achieved acceptable performance but showed inferior calibration to XGBoost in certain risk ranges. Compared with conventional logistic regression, XGBoost yielded higher net benefit across most clinically relevant threshold values and demonstrated superior value for perioperative risk stratification (Figure 4).

## 4. Discussion

### 4.1. Treatment Protocol

Current standard management for PDA in preterm infants involves fluid restriction, diuretics, and COX inhibitors [7]. However, COX inhibitors have contraindications (e.g., gastrointestinal bleeding) and fail to close the PDA in 15–30% of cases [8]. Prolonged preoperative waiting time is associated with higher postoperative ventilator settings and longer MV duration [9]. Therefore, surgical ligation or transcatheter closure is necessary when medical treatment fails. Although transcatheter closure offers definite efficacy and low risk [10], interventional closure for preterm infants with PDA is only available in a limited number of centers. Meanwhile, the complication rate of surgical procedures is approximately 10% [11], making surgical PDA ligation a more common therapeutic option. Our institutional strategy follows this approach: two courses of COX inhibitors for eligible infants, followed by timely ligation if medical treatment fails or is contraindicated. In this study, the median operative age was 32 days, consistent with the international consensus on 4-week-postnatal PDA ligation in preterm infants. Surgery was advanced in some infants due to pulmonary hemorrhage, while failed long-term conservative treatment at external hospitals led to obvious surgical delay in transferred cases.

### 4.2. RSS

RSS is a key indicator of preoperative respiratory status. Studies showed that when RSS > 4, the risk of respiratory failure increases significantly, both preoperatively and postoperatively, and such infants are more vulnerable to HFV support [12,13]. Adrianne R Bischoff et al. [14,15] identified high preoperative RSS as an important variable associated with postoperative clinical instability, significantly prolonging postoperative MV. In this study, both logistic regression analysis and machine learning algorithms consistently indicated that the RSS is the most important factor associated with prolonged postoperative MV duration: an RSS greater than 4.5 was associated with a significantly increased likelihood of postoperative MV > 6 days, AUC = 0.865 (95% CI: 0.812–0.918). Preoperative PHA will inevitably lead to respiratory deterioration and elevated RSS. This likely explains the higher rates of preoperative HFV use, higher incidence of preoperative PHA, and higher RSS values observed in the prolonged MV group in our study. We also considered whether prior NEC surgery might prolong MV duration; however, no significant association was found, suggesting that gastrointestinal surgery itself does not have a substantial impact on respiratory outcomes.

### 4.3. Weight and Age

Multiple studies have demonstrated that lower gestational age, lower birth weight, and lower weight at surgery are associated with prolonged postoperative MV [16,17,18], and the findings of our study are consistent with these previous reports: both logistic regression analysis and machine learning algorithms identified low birth weight as a strong predictor for prolonged postoperative MV. Preterm infants with a birth weight ≤1500 g were at a significantly increased possibility of requiring MV for >6 days postoperatively, highlighting the need for careful preoperative optimization in this vulnerable population. The impact of age at surgery on postoperative MV duration remains a subject of debate. Some investigators suggested that younger age at ligation may increase the risk of postoperative PLCS, thereby prolonging MV [18], while others reported that early surgical intervention may reduce MV duration by mitigating PDA-induced lung injury [19]. In our study, we did not find a significant association between age at surgery and postoperative MV duration. We speculate that this may be because the effects of preoperative waiting time on infants are multifactorial, encompassing multiple systems, including respiratory and circulatory function, which could lead to heterogeneous clinical outcomes.

### 4.4. Postoperative Heart Rate

Reyin Lien et al. [20] reported that cardiac output decreases to 73% of baseline immediately following PDA ligation, recovering to approximately 92% within 24 h. Notably, stroke volume decreased only to 91%, suggesting that heart rate is a critical determinant of postoperative cardiac output. Therefore, in this study, we recorded heart rates during the first 24 h post-ligation and analyzed whether the slowest heart rate was associated with MV duration. Indeed, the prolonged MV group exhibited comparatively slower lowest heart rates compared to the short MV group (median 148 vs. 150 bpm; mean approximately 146 vs. 150 bpm; *p* = 0.02). Logistic regression analysis and machine learning algorithms confirmed that slower heart rate was a significant factor associated with prolonged postoperative MV. However, its predictive value alone was modest (AUC < 0.7), and it demonstrated greater predictive utility when combined with other factors.

### 4.5. Diameter of PDA

The degree of ductal shunting depends on PDA diameter and the aortopulmonary pressure gradient. Larger PDA diameter increases left-to-right shunting and pulmonary blood flow, leading to left heart dilation. The LA/AO ratio is therefore used to assess hemodynamic significance, with ratios > 1.4–1.5 often indicating need for surgical closure [21]. Krishnappa et al. [22] found that larger PDA diameter and greater shunt volume predicted shorter postoperative MV, suggesting greater respiratory improvement after ligation. However, our results contrast with this: larger PDA diameter, higher PDA/AO ratio, and bidirectional shunting were associated with prolonged MV. We propose that PLCS (AUC = 0.724) may explain this discrepancy. Larger PDAs are more likely to impair left ventricular function and reduce lung compliance preoperatively [23], and sudden ligation can precipitate acute afterload increase—both key triggers of PLCS, which we identified as an independent predictor.

## 5. Limitations

(1) Deceased patients were excluded from analyses, as these patients could not be weaned from mechanical ventilation, which may introduce survival bias. Nevertheless, only eight such cases were identified, and this small number exerted a negligible impact on our results. (2) Unblinded retrospective echocardiogram measurement may cause observer variation. We minimized this bias by having all measurements performed by a single experienced echocardiographer. (3) Continuous postoperative hemodynamic data were unavailable due to the retrospective analysis; postoperative heart rate was therefore used as an indirect surrogate for circulatory status. (4) This single-center cohort lacks external temporal or geographic datasets to validate our machine learning model, so the clinical utility of the model remains exploratory.

## 6. Conclusions

For preterm infants with contraindications to COX inhibitor therapy or in whom such treatment has failed, PDA ligation serves as a necessary intervention.

Higher RSS, lower birth weight, and slower postoperative heart rate are significant factors associated with prolonged MV. When an infant has a birth weight ≤ 1500 g or RSS > 4.5, combined with other risk factors, the likelihood of postoperative MV lasting more than 6 days increases markedly, warranting active optimization of the preoperative condition. Postoperative heart rate is an early marker of prolonged mechanical ventilation; levels below 135 bpm increase the likelihood of MV > 6 days.

## Figures and Tables

**Table 1:** Baseline characteristics (*n* = 271).

Variable	Value
Male	152 (56.1)
Gestational age (weeks)	27 (23–32)
Birth weight (g)	920 (470–2220)
Age at surgery (days)	32 (6–113)
Weight at surgery (g)	1500 (600–4400)
PDA diameter (mm)	2.9 (1.3–6.3)
PDA/AO	0.39 (0.18–0.9)
Increase in systolic blood pressure (%)	20.83 (2.13–96.55)
Increase in diastolic blood pressure (%)	33.33 (2.22–147.62)
Slowest heart rate (bpm)	149 (115–185)
RSS	5.4 (2.52–24.5)
Bidirectional shunting	58 (21.4)
NEC	58 (21.4)
HFV	36 (13.3)
PLCS	99 (36.5)
PHA	81 (29.9)
Postoperative MV duration (days)	6 (1–68)

Data are presented as median (range) for continuous variables and *n* (%) for categorical variables.

**Table 2:** Comparison of variables between short and prolonged mechanical ventilation groups.

Variable	Short MV Group (*n* = 150)	Prolonged MV Group (*n* = 121)	*p* Value	FDR q Value
Male	87 (58)	65 (53.7)	0.56	0.549
Gestational age (weeks) *	27 (24–32)	26 (23–32)	<0.0001	<0.001
Birth weight (g)	1035 (610–2220)	810 (470–1870)	<0.0001	<0.001
Age at surgery (days)	32 (7–113)	31 (6–109)	0.3	0.396
Weight at surgery (g) *	1700 (800–4300)	1300 (600–4400)	<0.0001	<0.001
PDA diameter (mm)	2.7 (1.5–5.4)	3 (1.3–6.3)	0.01	0.010
PDA/AO	0.38 (0.18–0.9)	0.39 (0.21–0.82)	0.38	0.474
Increase in systolic blood pressure (%)	21.32 (2.13–96.55)	20.83 (3.45–90.32)	0.93	0.975
Increase in diastolic blood pressure (%)	35.16 (5–147.62)	30 (2.22–111)	0.08	0.115
Slowest heart rate (bpm) *	150 (115–181)	148 (115–185)	0.02	0.039
RSS *	4 (2.52–10.8)	7.2 (2.52–24.5)	<0.0001	<0.001
Bidirectional shunting*	23 (15.3)	35 (28.9)	0.01	0.012
NEC	32 (21.3)	26 (21.5)	1	0.975
HFV *	8 (5.3)	28 (23.1)	<0.0001	<0.001
PLCS *	27 (18)	72 (59.5)	<0.0001	<0.001
PHA *	19 (12.7)	62 (51.2)	<0.0001	<0.001

All continuous variables were non-normally distributed and are presented as median (range). Categorical variables are presented as counts (%). * indicates statistical significance (*p* < 0.05).

**Table 3:** Logistic regression analysis of factors associated with prolonged MV.

Variable	Wald χ^2^	*p* Value	OR (95% CI)
RSS	32.19	<0.001	3.89 (2.26, 6.69)
PHA	11.74	0.001	1.90 (1.30, 2.77)
PLCS	4.63	0.031	1.47 (1.03, 2.09)
PDA/AO	4.19	0.041	1.46 (1.01, 2.11)
Negative birth weight	16.39	<0.001	5.26 (2.04, 13.55)
Negative slowest heart rate	4.66	0.031	1.59 (1.05, 2.39)

**Table 4:** ROC analysis of individual factors and the integrated model.

Variable	AUC (95% CI)	Cut-Off Value	Sensitivity (%)	Specificity (%)	Youden
RSS *	0.865 (0.812–0.918)	4.5	80.2	82.5	0.627
PHA	0.782 (0.715–0.849)	1	75.8	76.3	0.521
PLCS	0.724 (0.651–0.797)	1	71.3	70.8	0.421
PDA/AO	0.718 (0.645–0.791)	0.48	69.8	71.2	0.410
Negative birth weight *	0.843 (0.787–0.899)	1500	81.5	79.8	0.613
Negative slowest heart rate	0.685 (0.608–0.762)	135	67.5	68.2	0.357
Integrated Model *	0.910 (0.872–0.948)	0.423	81.8	86.0	0.678

* indicates excellent predictive performance.

**Table 5:** Sensitivity analysis of high RSS based on propensity score weighting.

Variable	Value
Definition of high RSS	RSS > 4.5
Number of cases in high RSS group	175
Number of cases in low RSS group	96
Proportion of MV > 6 days in high RSS group after weighting	55.8%
Proportion of MV > 6 days in low RSS group after weighting	14.0%
Weighted risk difference	41.8%
OR	7.75
95% CI	4.09–14.67
*p* value	<0.001
Effective sample size (ESS) after weighting	122.4
Maximum standardized mean difference (SMD) after weighting	0.146

**Table 6:** Predictive performance analysis of machine learning algorithms.

Model	Fold 1	Fold 2	Fold 3	Fold 4	Fold 5	Mean AUC	Std AUC	OOF AUC	Brier
LR	0.880	0.914	0.894	0.885	0.828	0.880	0.032	0.880	0.139
RF	0.903	0.900	0.896	0.912	0.839	0.890	0.029	0.885	0.151
SVM	0.875	0.893	0.865	0.857	0.830	0.864	0.023	0.863	0.151
XGBoost	0.913	0.935	0.915	0.893	0.849	0.901	0.033	0.896	0.130

## Data Availability

All data included in this study are available upon request by contact with the corresponding author.

## Supplementary Materials

The following supporting information can be downloaded at: https://www.mdpi.com/article/10.3390/jcm15134984/s1, File S1: Comparison of reintubation rates between short and prolonged Mechanical Ventilation groups.

## Abbreviations

The following abbreviations are used in this manuscript:

PDA	Patent ductus arteriosus
MV	Mechanical ventilation
COX	Cyclooxygenase
NICU	Neonatology intensive care unit
PHA	Pulmonary hemorrhage or atelectasis
NEC	Necrotizing enterocolitis
HFV	High-frequency oscillatory ventilation
RSS	Respiratory Severity Score
MAP	Mean airway pressure
FiO_2_	Fraction of inspired oxygen
PLCS	Post-ligation cardiac syndrome
