# Supplementary material for: Identifying Factors Associated with Prolonged Postoperative Mechanical Ventilation in Preterm Infants Undergoing Patent Ductus Arteriosus Ligation Using Machine Learning and SHAP Analysis: A Large-Sample Single-Center Retrospective Analysis
Source: J Clin Med. 2026 Jun 26;15(13):4984. doi: 10.3390/jcm15134984 (PMC13362179; doi:10.3390/jcm15134984)
Supplement: Supplementary file 1 [file jcm-15-04984-s001.zip › jcm-4349162-supplementary.pdf]

Supplementary File S1. Comparison of reintubation rates between short and prolonged Mechanical Ventilation groups

Group	Total cases	Number of reintubation cases	reintubation rate
Short MV group	150	4	2.7%
Prolonged MV group	121	40	33.1%

Fisher's exact test: $P < 0.001$; OR = 18.02, 95% CI: 6.23–52.19. Compared with patients with MV ≤ 6 days, those with MV > 6 days had a significantly higher rate of reintubation, indicating that the 6-day cutoff possesses clinical discriminative power in this cohort.
